# Contrasting Water Withholding Responses of Young Maize Plants Reveal Link Between Lipid Peroxidation and Osmotic Regulation Corroborated by Genetic Analysis

**DOI:** 10.3389/fpls.2022.804630

**Published:** 2022-07-06

**Authors:** Vlatko Galić, Selma Mlinarić, Matea Marelja, Zvonimir Zdunić, Andrija Brkić, Maja Mazur, Lidija Begović, Domagoj Šimić

**Affiliations:** ^1^Department of Maize Breeding and Genetics, Agricultural Institute Osijek, Osijek, Croatia; ^2^Department of Biology, Josip Juraj Strossmayer University of Osijek, Osijek, Croatia; ^3^Centre of Excellence for Biodiversity and Molecular Plant Breeding (CroP-BioDiv), Zagreb, Croatia

**Keywords:** GWAS, ontology, lipid peroxidation, proline, signaling, abiotic stress

## Abstract

Linking biochemistry and genetics of tolerance to osmotic stress is of interest for understanding plant adaptations to unfavorable conditions. The aims of this study were to investigate the variability in responses of panel of elite maize inbred lines to water withholding for stress-related traits through association study and to identify pathways linked to detected associations for better understanding of maize stress responses. Densely genotyped public and expired Plant Variety Protection Certificate (ex-PVP) inbred lines were planted in controlled conditions (16-h/8-h day/night, 25°C, 50% RH) in control (CO) and exposed to 10-day water withholding (WW). Traits analyzed were guaiacol peroxidase activity (GPOD), total protein content (PROT), lipid peroxidation (TBARS), hydrogen peroxide accumulation (H_2_O_2_), proline accumulation (proline), and current water content (CWC). Proline accumulation was found to be influenced by H_2_O_2_ and TBARS signaling pathways acting as an accumulation-switching mechanism. Most of the associations detected were for proline (29.4%) and TBARS (44.1%). Gene ontology (GO) enrichment analysis showed significant enrichment in regulation of integral membrane parts and peroxisomes along with regulation of transcription and polysaccharide catabolism. Dynamic studies involving inbreds with extreme phenotypes are needed to elucidate the role of this signaling mechanism in regulation of response to water deficit.

## Introduction

One of the key targets of maize (*Zea mays* L.) breeding is tolerance to abiotic stress conditions. Phenotyping for stress responses represents the key for success in breeding while the underlying trait physiology mostly remains unclear (Masuka et al., 2012). Plants adapt to sub-optimal conditions by morpho-physiological adjustments, with vast number of mechanisms on different organizational levels at their disposal (Pareek et al., 2010). However, genotypic variability for these adjustments exists and some genotypes are expected to cope with abiotic stress conditions better than others (Slafer and Araus, 2007; Tardieu, 2012). Water deficit represents one of the main abiotic stresses in the field conditions in rain-fed areas and can affect the plant growth and development at any time from emergence to yield formation causing an outburst of physiological responses (Wang et al., 2019). For example, water deficit affecting the plant during reproductive stages can cause the formation of smaller number of kernels or kernel abortion. In grain filling, it results in smaller grains and premature senescence, whereas the effects of drought in early plant development received relatively less attention despite the fact that water deficit at this stage can cause within-field variability in plant size, deteriorate the stands, and make the crop more susceptible to diseases (Farooq et al., 2012; Aslam et al., 2015). Climate change causes a significant alteration of spatiotemporal patterns of drought occurrence (Stagge et al., 2017; Grillakis, 2019), with more recent droughts lasting longer and occurring at less predictable times. Water deficit results in a number of adverse effects such as reduction of plant turgidity, reactive oxygen species (ROS) build-up, decrease in photosynthetic efficiency, and ultimately, the plant death. However, the plant mechanisms to cope with these adverse effects include the osmotic adjustments by synthetizing the osmotically active compounds and increase in enzymatic activity to detoxify the effects of ROS (Anjum et al., 2017).

During the oxidative stress periods, plant cell suffers damage at many levels, i.e., outer cell layers, cytoplasmic components, nucleus, and nucleic contents, inevitably leading to selective cell death. ROS exert highly oxidative cell surroundings, interacting with lipids (primarily polyunsaturated fats, PUFAs), proteins, and nucleic acids often resulting in limitations to biological yield (Czarnocka and Karpiński, 2018). One family of products of this highly detrimental interaction is the products of lipid biomolecule peroxidation called malondialdehydes (MDAs). MDAs are highly reactive in oxidative surroundings expected in oxidative stress due to their α-β-unsaturated carbonyl group and are thus known as reactive carbonyl species (RCS). The well-known instability and reactivity of these species makes it unfeasible to measure them directly, so the products of their secondary activity, reacting with thiobarbituric acid (i.e., *thiobarbituric acid reactive substances*, TBARS), are measured instead. RCS are in parallel produced both enzymatically (lipoxygenase activity) and non-enzymatically (ROS-mediated), and both processes also occur in healthy organisms (Farmer and Mueller, 2013). However, temporary increase in MDA levels in stress conditions represents acclimation process that activates regulatory gene networks involved in plant defense and development such as dehydration/heat shock-related genes and genes involved in antioxidant machinery (Morales and Munné-Bosch, 2019). Moreover, MDA can cause the transcriptional reprogram of a cell, activating transcription of abiotic-stress related genes, making them effective signal molecules (Weber et al., 2004). Another versatile plant signal molecule is hydrogen peroxide (H_2_O_2_). H_2_O_2_ is a ROS byproduct of metabolism, mostly built-up during stress-induced respiratory burst of plant plasma-membrane NADPH oxidases by superoxide-dismutase from more toxic oxygen species, or from the process of β-oxidation of lipid molecules in membrane bound microbodies peroxisomes (Corpas et al., 2020). In other cell compartments, lower doses of H_2_O_2_ show a limited cellular toxicity, as it is easily accumulated in plant cells by downregulation of its peroxisome-localized degradation enzymes such as catalase, ascorbate peroxidase, glutathione peroxidase, and so on. Which makes for a robust signaling molecule (Hossain et al., 2015). Generally, the H_2_O_2_ degradation process in maize is carried out by two classes of peroxidases; the ones utilizing its substrate in lignification and organogenesis, such as guaiacol peroxidases, and the others scavenging the peroxide molecules utilizing pyridine nucleotides, GSH, cytochrome *c* and ascorbate as electron donors (Prasad et al., 1995). The former group is involved in the young plant development, whereas both groups are involved in stress responses (Gechev et al., 2006) and signaling (Kidwai et al., 2020). One of the main tasks of H_2_O_2_ in stress-signaling appears to be the regulation of osmolyte synthesis, specifically proline, through transcriptional upregulation of proline-biosynthesis genes (Yang et al., 2009), and downregulation of its degradation pathways. Since the downregulation of peroxisomal H_2_O_2_-scavenging enzymes appears to be the main source of signaling H_2_O_2_ in cells (Su et al., 2019), and the peroxisomes also serve as the alternative cell energy supply by lipid catabolism, it is possible that by alterations of the peroxisomal regulation, some other signaling cascades become dominant or more pronounced.

Maize breeding relies on several germplasm resources (Lee and Tracy, 2009), key of which is the elite germplasm available after the expiration of plant variety protection (PVP) certificate, the so-called ex-PVP germplasm (Mikel and Dudley, 2006). In modern maize breeding, with maize holding the majority of world seed market (FAO/IHS Markit Agribusiness Consulting, 2019), the ex-PVP germplasm still prevails the new inbred registrations (Mikel, 2011; White et al., 2020). This extremely valuable germplasm resource consists of thousands of genotypic accessions (Romay et al., 2013) with traceable pedigrees and available comprehensive genotypic and phenotypic data (Canaran et al., 2008). Many studies were conducted based on this resource; however, studies combining the physiological assessment with dense genotypic data in elite germplasm are scarce. Moreover, the studies reporting results of association analysis for traits assessing oxidative status, lipid peroxidation, and proline accumulation in maize are scarce. Breeding for tolerance to early osmotic stress might be a meaningful endeavor due to the adverse effects of stress at this stage on stands and crop health status (Farooq et al., 2012; Aslam et al., 2015), corroborated by high variability of available germplasm resources in terms of ancestry (Lee and Tracy, 2009), genotype (White et al., 2020), and consequently the phenotype (Galić et al., 2020). It was hypothesized that integration of data assessing plant osmotic status (CWC, proline, PROT), plant oxidative status (TBARS, H_2_O_2_), and dense genotypic data might enhance detection of new important loci or pathways for adaptation to osmotic stress, as well as to highlight the gene ontology (GO) enrichment, thus facilitating the discovery of new regulatory networks involved in plant response to water withholding. An enrichment analysis of GO is an efficient methodology for the assessment of functions linked to large gene lists increasing the likelihood of interpretation of the detected biological processes and regulatory networks (Tian et al., 2017). In the advent of high-throughput molecular techniques identifying more and more genes and generating the big data, combining GO with association mapping helps to bridge the gap in translation of genomes to phenomes (Pan et al., 2019).

Based on that, the aims of this study were to investigate the contrasts in responses of panel of diverse, elite ex-PVP and public maize inbred lines in proline accumulation lipid peroxidation and oxidative status to water withholding in early stages of growth and combine these results with dense genotypic data to increase precision and true detection rate in association analysis. We further aimed to establish the relationships between the assessed phenotypic traits and use the results of genetic analysis to determine the enriched biological processes involved in maize osmotic regulation. Our study represents a novel effort in maize to corroborate the findings in stress biochemistry with enrichment analysis of underlying genetic associations in a panel of elite inbred lines with global significance.

## Materials and Methods

### Plant Material and Experimental Design

The experimental design was previously described in Galić et al. (2020). Briefly, seeds of maize inbred lines were freely collected according to the US Plant Variety Protection Act upon the expiration of their respective certificates from US National Plant Germplasm System (NPGS) according to their Distribution Policy (https://npgsweb.ars-grin.gov/gringlobal/distribution, accessed May 5, 2022). Seeds were transferred with their enclosed passport documents and USDA-APHIS Plant Export and Phytosanitary Certificates in fall of 2017. The maize inbred lines used to carry out this research were not subjected to any form of modification. Seeds were planted in field in growing season 2018 and selfed to obtain seeds in sufficient quantity for experiments. Selfing was successful for 109 inbreds. Experiments were conducted with awareness of the requirements the IUCN Policy Statement on Research Involving Species at Risk of Extinction and the Convention on the Trade in Endangered Species of Wild Fauna and Flora with intention to comply with all relevant institutional, national, and international guidelines and legislation. Experiments were set in controlled conditions (25°C, RH = 50%, 16-h/8-h day/night, 200 μmol/m^2^/s) in trays (510 mm × 350 mm × 200 mm) divided to 15 rows with 7 boxes (50 mm × 35 mm). Each tray was filled with 8.67 kg (20 L) of air-dry soil [pH (CaCl_2_) = 5.7, N (NH${}_{4}^{+}$ + NO${}_{3}^{-}$) = 70 mg/L, P (P_2_O_5_) = 50 mg/L, K (K_2_O) = 90 mg/L, EC = 40 mS/m] and the planting was performed to 2 cm depth. The experiment was set with single water withholding treatment (WW) and control (CO). A total of three biological replicates (trays) of each genotype were planted, with one tray containing a single biological replicate of 15 genotypes (rows), with single row (7 planting boxes) each to enable shuffling. Watering regime for WW was optimized in preliminary trials to obtain the 50% reduction in fresh weight per plant in WW treatment compared to CO. Plants in CO were watered with spray bottle in planting and every 2 days thereafter with 8 ml of tap water per plant. Amount of water added to CO was determined by weighting the soil after 3-day drainage following complete saturation of soil with water (field water capacity). Plants in WW were watered in planting with full dose (8 ml) and two times thereafter (last watering on 4th day) with half a dose of water added per plant in C (4 ml). After that, water was withheld up to the 14th day since planting (10 days of water withholding) when the aboveground parts of three equally developed plants per genotype in each replicate were harvested. A total of eight trays were left without plants in the same conditions for soil weighting. Trays were treated in the same way as the experimental ones, with four trays representing Co and the other four representing WW. Trays were weighted every day. The 1 g samples were taken for analysis of current water content (CWC), whereas the rest was frozen on liquid nitrogen and left in −80°C freezer for further analyses. Tubes with 1 g samples were dried for 24 h in digital laboratory oven on 80°C. Current water content (CWC) was calculated:


(1) $$\begin{array}{r} CWC=\frac{Fresh\;weight-Dry\;weight}{Fresh\;weight}\;*\;100 \end{array}$$

### Biochemical Analyses

All biochemical analyses were carried out in three biological replicates, each further measured in three technical (lab) replicates. Proline content (proline) was determined according to Carillo and Gibon (2011). About 20 mg of fresh seedling tissue was extracted in 400 μl (ethanol: water, 40:60 v/v) overnight at 4°C. For measurements, 50 μl of extract was used. Measurements were taken on microplate reader (Tecan, Spark) at 520 nm. Proline content was calculated from the standard curve using proline as standard and expressed as nmol/mg of fresh weight (FW).

Analysis of TBARS (thiobarbituric acid reactive substances) was performed according to the method described by Jambunathan (2010). After tissue homogenization in liquid nitrogen, about 0.2 g of plant tissue was extracted by the addition of 1 ml of 0.1% trichloroacetic acid (TCA). The samples were centrifuged for 5 min at 6,000 g at 4°C. After centrifugation, 0.5 ml of the supernatant was separated into a screw cap tube and 1 ml of TBA in TCA (0.5% thiobarbituric acid solution in 20% trichloroacetic acid solution) was added. The blank contained 1.5 mL of TBA in TCA. The reaction mixture was vortexed and incubated in a water bath for 30 min at 95°C followed by centrifugation for 15 min at 18,000 rpm at 4°C. The absorbance was measured at 532 and 600 nm. Obtained results were expressed as nmol/g of fresh weight (FW).

Concentration of H_2_O_2_ was determined by the method according to Mukherjee and Choudhuri (1983). After tissue homogenization in liquid nitrogen, 0.1 g of powder was extracted with 1 ml of cold acetone. The reaction mixture was vortexed and centrifuged for 5 min at 6,000 g and 4°C. The supernatant was separated, and 400 μl of titanium sulfate solution and 500 μl of concentrated ammonium hydroxide (NH_4_OH) were added. The reaction mixture was centrifuged at 15,000 rpm for 10 min at 4°C. The supernatant was decanted and the resulting precipitate was dissolved by the addition of 1 ml of 2M H_2_SO_4_ solution. The absorbance was measured at 415 nm, and the H_2_O_2_ concentration was expressed as nmol/g of sample fresh weight (FW).

The method described by Siegel and Galston (1967) for determination of guaiacol peroxidase (GPOD) was adapted for microplate reader. Briefly, approximately 0.2 g of previously powdered seedling tissue was extracted with 1 ml of 0.1 M phosphate buffer (pH 7.0). Samples were centrifuged at 18,000 rpm at 4°C. After centrifugation, samples were re-extracted and supernatants were pooled. Reaction mixture was prepared using 8 mM H_2_O_2_ and 90 mM guaiacol in 1:1 ratio (v/v). All measurements were taken in triplicates in 96-well plates using microplate reader (Tecan, Spark) by adding 150 μl of phosphate buffer, 40 μl guaiacol/H_2_O_2_ mix, and 10 μl of extract. Absorbance was read at 436 nm using extinction coefficient of 25.5 mM^−1^cm^−1^, and results were expressed as units per gram of sample fresh weight (U/FW).

Total protein content (PROT) in the samples was determined by the Bradford method (Bradford, 1976) adapted for microplate reader. Proteins were measured in same extract used for peroxidase activity assay. Briefly, 5 μl of sample and 250 μl of Bradford reagent were mixed and absorbance was read at 595 nm. The preparation of standard curve dilutions of bovine serum albumin (BSA) was used (0.125–1.4 mg/ml). The protein concentration was expressed as mg/g fresh weight.

### Analyses of Phenotypic Traits

Pearson's product-moment correlations were calculated between genotypic means of all traits to establish trait connections within as well as between control and WW treatment. Genotypic means represented a mean value of three technical replicates over three biological replicates, totally 9 data points per genotype in each treatment. For each trait, fold-change values were calculated to examine specific patterns of reactions as $Tr_{rel}=\frac{Tr_{ww}-Tr_{co}}{Tr_{co}}$, where *Tr*_*rel*_ represents trait fold-change in water withholding (*Tr*_*ww*_) relative to trait value in control conditions (*Tr*_*co*_). Correlations were also calculated between trait fold-changes to establish connections between different physiological indicators. Correlation coefficients were displayed using the *R/gplots* package (Warnes et al., 2013) function *heatmap.2*. Relationships between traits were imposed using a correlation distance-based clustering.

To inspect the distinct patterns of reactions between groups of genotypes, unsupervised K-means clustering analysis was carried out in R/*factoextra* library (Kassambara and Mundt, 2020) with *Tr*_*rel*_ values as input. To determine the optimal number of clusters, a Silhouette statistic was computed (Charrad et al., 2014), and the optimal number of clusters was 2 (Supplementary Figure S1).

Variance components of all traits were calculated in R/*sommer* library (Covarrubias-Pazaran, 2016). Models were specified with unstructured error variances as follows:


(2) $$\begin{array}{r} y_{ijk}=G_{i}+\left(T_{j}\right)+GT_{ij}+\varepsilon_{ijk} \end{array}$$

where *y*_*ijk*_ was value of *i*-th genotype in *j*-th treatment in *k*-th replicate, *G*_*i*_ represents random effects of genotype, (*T*_*j*_) represents fixed treatment effects, *GT*_*ij*_ is the random genotype-treatment interaction term, and ε_*ijk*_ is the overall model error term. Estimates of errors of variance components and the trait repeatabilities were calculated in package's *pin* calculator. Trait repeatabilities were calculated as: $H^{2}=\frac{\sigma_{G}^{2}}{\sigma_{G}^{2}\;+\;\frac{\sigma_{GxT}^{2}}{n_{t}}+\frac{\sigma_{\varepsilon }^{2}}{n_{t}*n_{r}}}$, where $\sigma_{G}^{2}$, $\sigma_{GxT}^{2},$ and $\sigma_{\varepsilon }^{2}$ represent variance components of genotype, genotype by treatment interaction and error, respectively, whereas *n*_*t*_ is the number of treatments and *n*_*r*_ is the number of replicates. Satterthwaite's estimates of *p*-values for differences between treatment effects were calculated in R/*lmerTest* library (Kuznetsova et al., 2017).

### Genotypic Data Manipulation and Analysis

Genotyping was performed at Cornell University with genotyping by sequencing approach with protocol from Elshire et al. (2011). Genotypic data were the part of the genotyping efforts of the US National Plant Gemplasm System Gene Bank consisting of tens of thousands of maize accessions (Romay et al., 2013). The genotypic data were retrieved from Panzea (panzea.org) repository (Canaran et al., 2008) as partially imputed calls with ~955,000 SNP positions on AGPv4 B73 reference alignment. Dataset 1 was constructed by filtering the original partially imputed GBS data to maximum 10% missing data, minor allele frequency of 0.02, and no heterozygotes. Filtering resulted in 107,527 positions. Positions were imputed by using LinkImpute methodology (Money et al., 2015) with 30 sites in high LD and 10 nearest neighbors. For Dataset 2, LD pruning of positions was performed in Plink 1.9 (Purcell et al., 2007) with –indep 50 5 0.95 flag to prevent false positives in association analysis. Pruning resulted in 70,130 variants. Dataset 2 was used for PCoA, kinship, and association analysis. Association analysis was performed in mixed-effects linear model framework (MLM+Q+K) with Q matrix calculated in principal coordinate analysis with 7 assumed axes and identity-by-state kinship matrix (K) as covariate in Tassel software (Bradbury et al., 2007) version 5.2.67. To further control false detection rate (FDR), cluster affiliations of different genotypes from K-means analysis were used as covariate. The *Tr*_*rel*_ values were used as phenotypes in association analysis. Arbitrary –log(p) threshold of 4 was used to declare significant associations according to the results of Bian and Holland (Bian and Holland, 2017) that showed the stable predictive abilities of the loci detected in the range of –log(P) thresholds from value of 4 to Bonferroni-corrected value in oligogenic and polygenic traits. Bonferroni threshold was also determined following the simpleM procedure described by Gao et al. (2008, 2010). Briefly, of the 70,130 filtered and imputed markers, the effective number of markers (M-_eff_) was determined to be 13,966 and the significance threshold of α = 0.05 was divided with the M_eff_ which resulted in –log(p) value of 5.446.

The scan for genes associated with detected positions was carried out using a Ensembl Plants service (Howe et al., 2020), BioMart tool (Kinsella et al., 2011). The scan was limited to protein-coding genes 120 kbp from the detected associations in both directions, according to the results of linkage disequilibrium in this association panel (Galić et al., 2020). Only the protein-coding genes were analyzed. All 182 detected genes were subjected to AgriGo version 2.0 analysis for enrichment of biological processes, pathways, and cellular components (Tian et al., 2017), but only 162 genes with known pathways were analyzed. AgriGo uses the Fisher's test and the *z-scores* to retrieve the enriched terms, taking into the account total number of one organism's genes annotated with GO or of the user-provided background, the number of genes mapped to the background in the query list, the total number of genes in one GO term and the counts of overlapped genes as well as the means, and standard deviations of sample counts. The software's acyclic drawer is based on semantic similarity measurement (SSM) described in Wang et al. (2007).

## Results

### Treatment Effects and Variance Components

Water withholding treatment induced visible drying of soil in terms of field water capacity (FWC). On the day 0 (last watering in WW), the mean soil water content in WW was 75.4% compared to 90.1% in CO. The difference was induced by adding only a half dose of water to the soil in WW compared to CO at day 0. On the day of sampling (day 10), the mean soil water content dropped to 22.8% FWC (Supplementary Figure S2). In CO, the soil water content was maintained between 72.5 and 93.2% FWC. Water withholding treatment induced significant changes in all measured biochemical traits (Table 1) except PROT (*p* = 0.362). TBARS was in average significantly increased in WW treatment compared to control (48.5% increase), as well as proline (288.1%), H_2_O_2_ (23.5%), and GPOD (6.38%). Significant decrease was observed only in CWC (2.61%). Non-zero estimates of variance components were observed for GPOD, TBARS, proline, H_2_O_2_, and CWC (Table 1). Genotype by treatment interaction was larger than genotypic variance only in proline and TBARS. All repeatability estimates were larger than zero, spanning from 0.28 (proline) to 0.80 (H_2_O_2_).

**Table 1:** Mean values ± standard deviations and *p*-values of differences in means in control (CO) and 10-day water withholding treatment (WW) for GPOD (AU/g FW) PROT (mg/g FW), TBARS (ng/g FW), proline (nmol/mg FW), H_2_O_2_ (nmol/g FW), and CWC (%) followed by variance components and repeatabilities ± standard errors of repeatabilities and the results of K-means clustering analysis with cluster means of trait fold-change and the accompanying *p*-values of pairwise *t*-tests between cluster means.

	**GPOD**	**PROT**	**TBARS**	**Proline**	**H_**2**_O_**2**_**	**CWC**
**Treatment effects**						
CO	0.94 ± 0.23	0.72 ± 0.21	4.14 ± 1.18	1.85 ± 0.66	1.66 ± 0.55	93.52 ± 0.85
WW	1.00 ± 0.29	0.67 ± 0.25	6.15 ± 2.00	5.33 ± 3.55	2.05 ± 0.71	90.92 ± 1.67
*p*-value	0.00808	0.362	<0.001	<0.001	<0.001	<0.001
**Variance components and repeatabilities**						
$\sigma_{\text{G}}^{2}$	0.036	1.404	1.181	1.068	0.269	0.862
$\sigma_{\text{GxT}}^{2}$	0.028	0.998	1.461	5.360	0.117	0.627
$\sigma_{\text{e}}^{2}$	0.014	0.467	0.193	0.263	0.049	0.781
*R^2^*	0.68 ± 0.06	0.71 ± 0.06	0.61 ± 0.08	0.28 ± 0.14	0.80 ± 0.04	0.66 ± 0.07
**K-means cluster analysis**						
Cluster 1 (*n* = 65)	0.010 ± 0.22	−0.002 ± 0.421	0.321 ± 0.265	0.982 ± 0.691	0.188 ± 0.299	−0.312 ± 0.152
Cluster 2 (*n* = 44)	0.195 ± 0.37	−0.107 ± 0.324	0.863 ± 0.517	3.400 ± 2.475	0.407 ± 0.353	−0.545 ± 0.243
*p*	0.003964	0.1453	<0.001	<0.001	0.001127	<0.001

### Correlation Analysis

**Figure 1:**
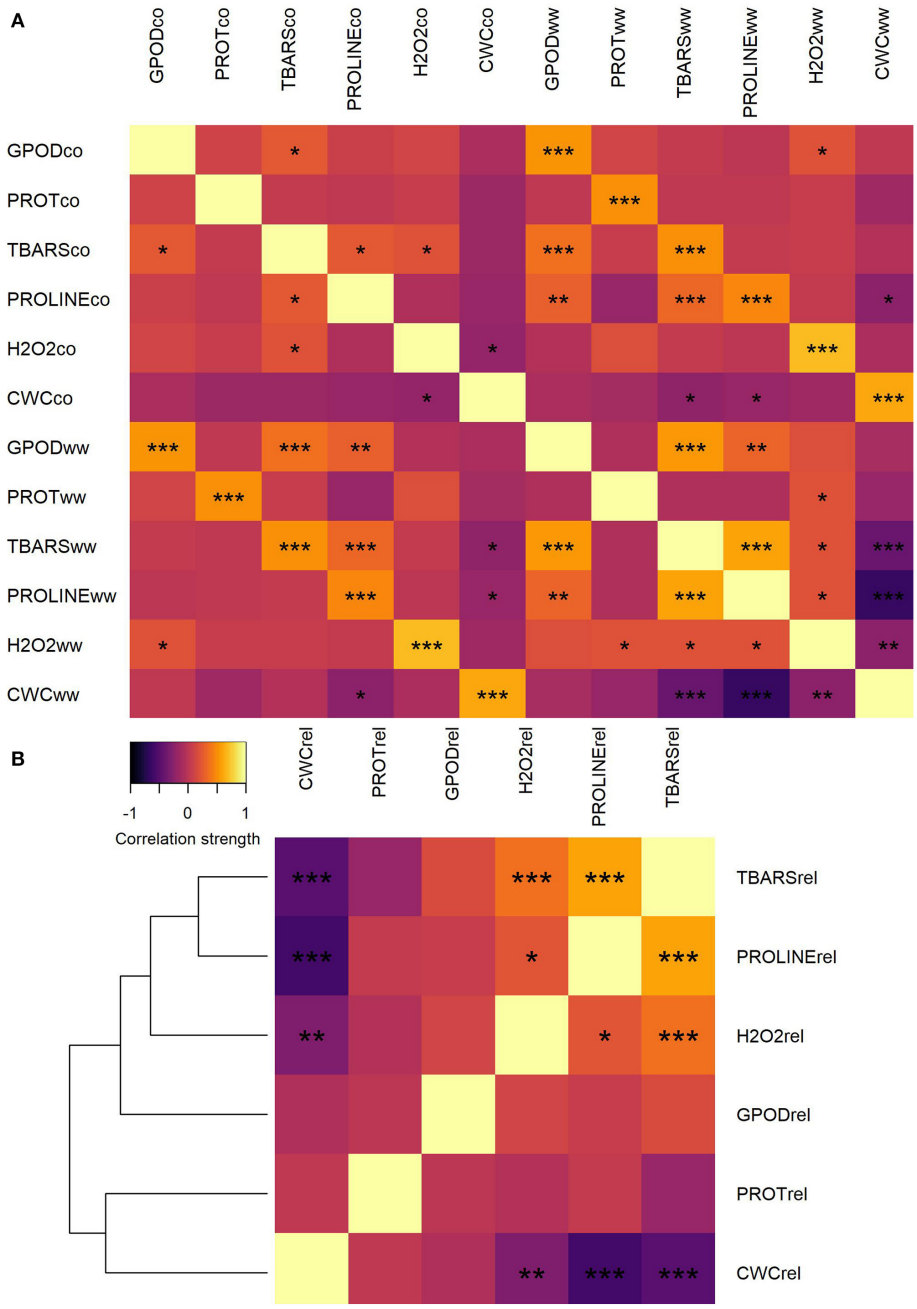
Heatmap of correlations between trait mean values in CO and WW **(A)** and heatmap and correlation-distance cladogram of correlations between trait fold-change values in WW compared to CO **(B)**. *, **, and *** represent significance of correlations at α = 0.05, α = 0.01, and α = 0.001 levels, respectively.

Correlation analysis of trait values between CO and WW treatment showed moderate to strong, significant correlations between all traits (Figure 1A). Lowest correlation between CO and WW was observed for proline (0.455, not shown), whereas the highest was observed for H_2_O_2_ (0.690). Very weak to weak positive and negative correlations were detected between all traits in CO, whereas in WW, most of the correlation strengths increased. Moderate positive correlation was observed in WW between GPOD and TBARS (0.555) and between TBARS and proline (0.595). Contrarily, the strong negative correlation was observed in WW between CWC and proline (−0.637), whereas the correlation between TBARS and CWC was moderate negative (−0.404).

Trait fold-change was calculated and used for further analyses because the change in the assessed parameters is of true interest for understanding plant adaptation to osmotic stress conditions and differentiate sensitive genotypes from tolerant ones. When the correlations were calculated between trait fold-changes, clustering of traits by correlation patterns was observed (Figure 1B). A number of two separate clusters were formed: one with TBARS, proline, H_2_O_2_, and GPOD and another with PROT and CWC. Strongest positive correlation was observed between fold-change values of TBARS and proline (0.583). Significant weak positive correlations were also detected between H_2_O_2_ and TBARS (0.368) and between H_2_O_2_ and proline (0.220). Strongest negative correlation was observed between fold-change values of proline and CWC (−0.578). Other two significant negative correlations were detected between fold-change values of CWC and TBARS (−0.477) and between CWC and H_2_O_2_ (0.300).

### Crossover Reactions Indicate Involvement of TBARS in Osmotic Stress Signaling

**Figure 2:**
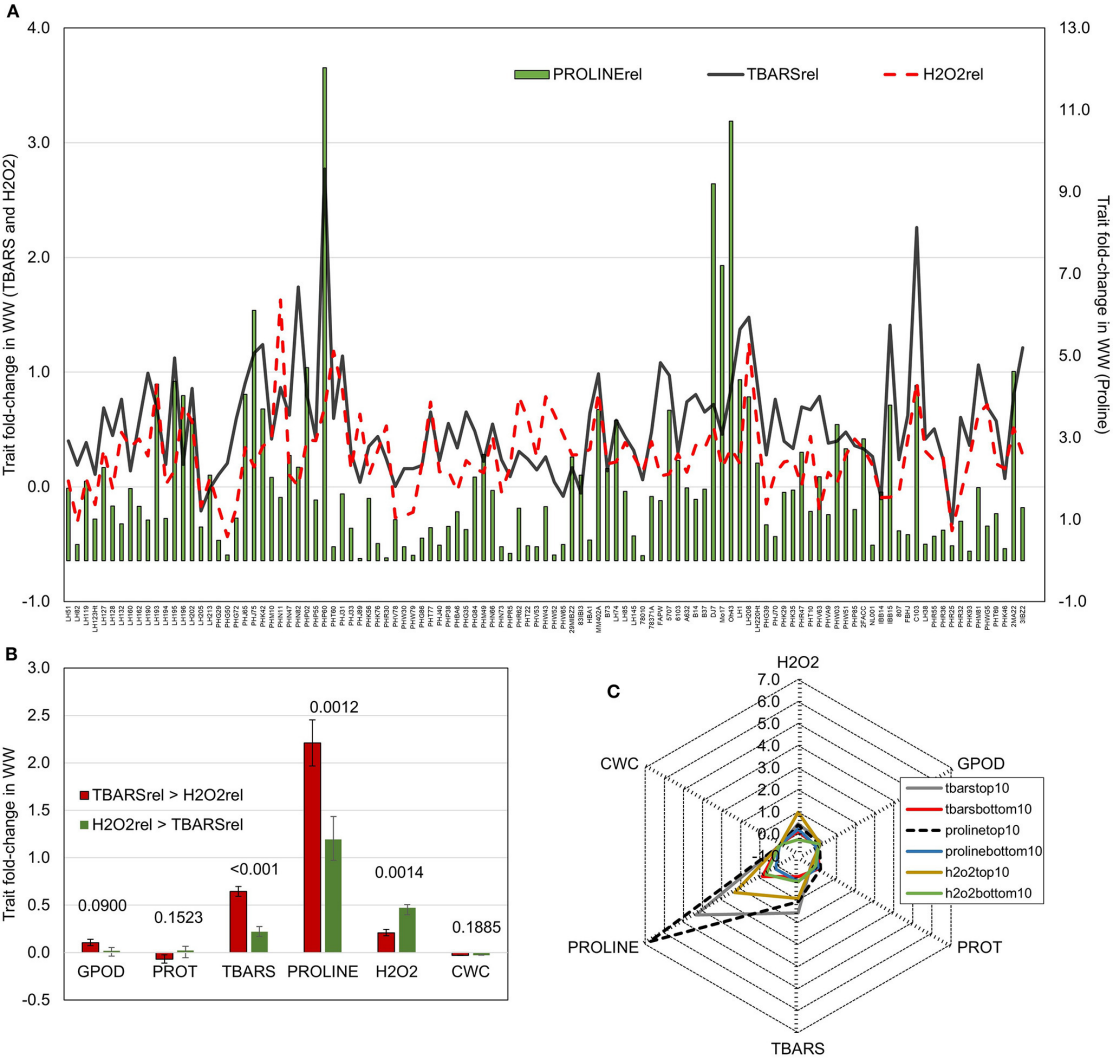
**(A)** Fold-change in WW compared to CO of TBARS and H_2_O_2_ accumulation (left y-axis) and proline (right y-axis); **(B)** Fold changes in WW compared to CO of GPOD, PROT, TBARS, proline, H_2_O_2_, and CWC in groups with larger fold change of TBARS than H_2_O_2_ (TBARS > H_2_O_2_) and with larger fold change of H_2_O_2_ than TBARS (H_2_O_2_ > TBARS). *p*-values of pairwise *t*-tests between groups are given above bars; **(C)** radar-plots of fold changes WW compared to CO of GPOD, PROT, TBARS, proline, H_2_O_2_, and CWC in top and bottom 10 performers in fold change of TBARS, proline, and H_2_O_2_.

To further analyze the relationship patterns of reactions in lipid peroxidation, hydrogen peroxide build-up, and proline accumulation, trait fold-changes were plotted on a 2-y-axis plot (Figure 2A). Highest values of proline accumulation were observed in accessions where TBARS fold-change crossed the value of fold-change in H_2_O_2_. Contrarily, in genotypes in which fold-change in H_2_O_2_ was larger than the fold-change in TBARS, proline accumulation appeared to be lower. Genotypes were divided in two groups following this pattern, the one in which fold-change of TBARS was larger than fold-change in H_2_O_2_ (TBARS > H_2_O_2_), and another in which fold-change in H_2_O_2_ was larger compared to fold-change in TBARS (H_2_O_2_ > TBARS). The analysis showed significantly larger fold-change in TBARS (0.645 vs. 0.221) and proline (2.21 vs. 1.19) in TBARS > H_2_O_2_ group, and significantly lower fold-change in H_2_O_2_, whereas other analyzed traits were not significantly affected (Figure 2B). Moreover, analysis of the top 10 scorers for proline, TBARS, and H_2_O_2_ fold-change revealed that highest mean fold-change in TBARS was accompanied by second-highest fold-change in proline accumulation of 4.35 (after top 10 scorers for proline with mean of 6.77), followed by top 10 scorers in H_2_O_2_ with mean fold-change in proline of 2.31 (Figure 2C).

Most interestingly, correlation analysis of trait fold-changes between two groups (TBARS > H_2_O_2_ and H_2_O_2_ > TBARS) showed strong positive significant correlation between H_2_O_2_ and TBARS in H_2_O_2_ > TBARS group (Table 2), compared to moderate significant correlation between these traits in TBARS > H_2_O_2_ group, indicating possible activation of the alternative pathway of MDA build-up. The increase in correlation strength from non-significant weak in H_2_O_2_ > TBARS to moderate to strong significant positive in TBARS > H_2_O_2_ group indicated possible involvement of this increase in lipid peroxidation in proline accumulation signaling.

**Table 2:** TBARS (ng/g FW), proline (nmol/mg FW) and H_2_O_2_ (nmol/g FW) means ± standard errors of mean in WW compared to CO, and the correlations (bold) between the traits in two contrasting groups (Figure 5A), TBARS > H_2_O_2_, and H_2_O_2_ > TBARS.

	**Group**	**TBARS**	**PROLINE**	**H_**2**_O_**2**_**
CO	TBARS > H2O2	3.958 ± 0.125	1.828 ± 0.074	1.7 ± 0.064
	H2O2 > TBARS	4.698 ± 0.23	1.917 ± 0.124	1.535 ± 0.084
WW	TBARS > H2O2	6.324 ± 0.23	5.684 ± 0.403	1.994 ± 0.074
	H2O2 > TBARS	5.622 ± 0.317	4.244 ± 0.583	2.216 ± 0.153
**PROLINE**	**TBARS > H2O2**	***0.590***	**–**	**–**
	**H2O2 > TBARS**	**0.181**	**–**	**–**
**H**_**2**_ **O**_**2**_	**TBARS > H2O2**	***0.564***	***0.391***	**–**
	**H2O2 > TBARS**	***0.812***	**0.118**	**–**

*Correlations significant at α = 0.05 level are shown in italic*.

**Figure 3:**
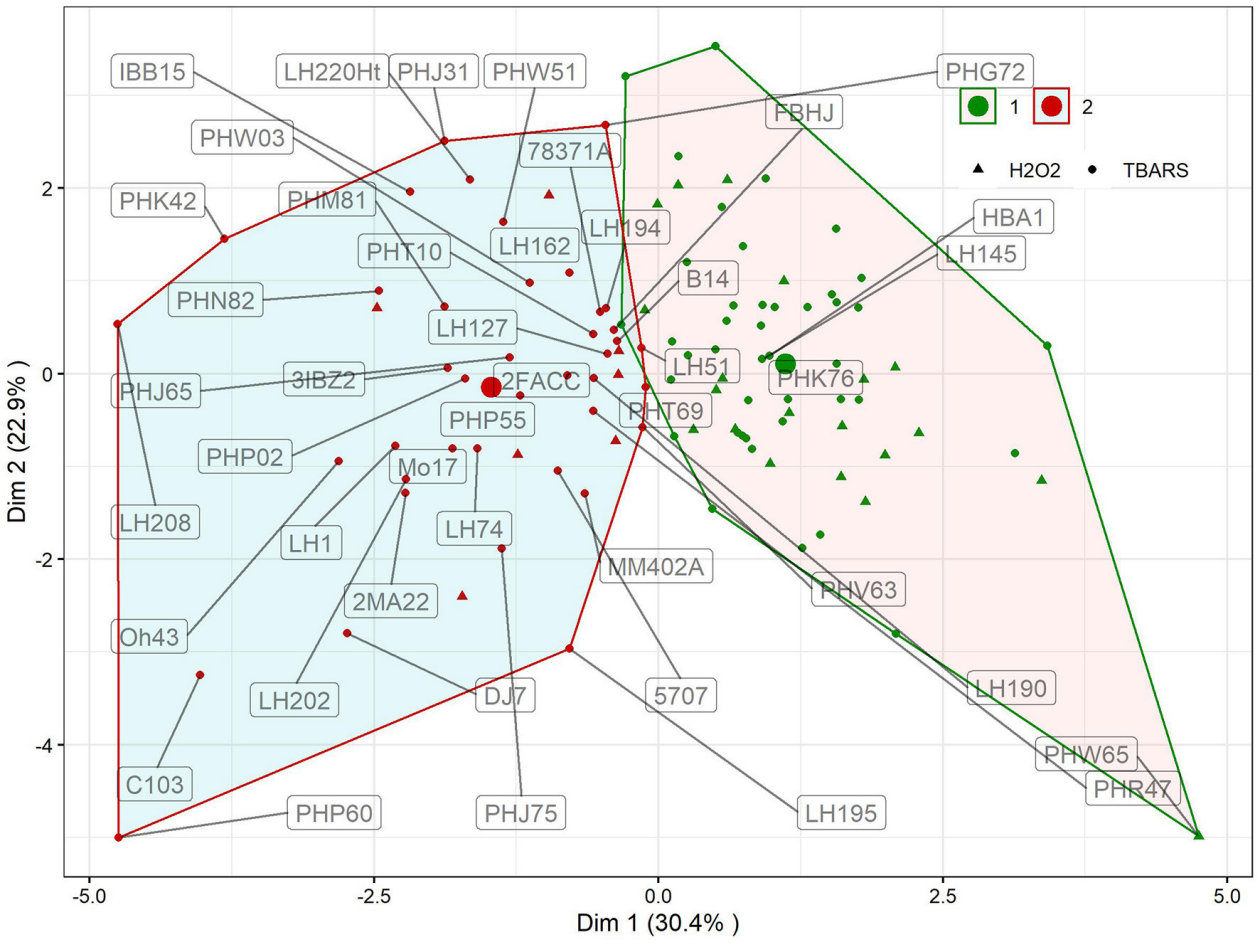
Results of K-means clustering of genotypic means using trait fold-change values of GPOD, PROT, TBARS, proline, H_2_O_2_, and CWC in WW compared to CO. Inbred designations are given for all inbreds belonging to the cluster 2 and TBARS > H_2_O_2_ group, along with inbreds surrounding the centroid in cluster 1 and the most extrema phenotype in cluster 1 (PHW65). Large dots show centroid of each cluster.

K-means clustering with trait fold-change showed two distinct clusters of reactions (Figure 3). Clustering explained 53.3% of total variation in first two dimensions. In first cluster, 65 genotypes with moderate reactions to WW treatment were grouped, whereas in the other, the reactions of 44 genotypes to WW were more pronounced. Cluster designations of inbreds are available in Supplementary Table S1, available online. Differences in changes in reactions between clusters were significant for all traits except PROT (Table 1, *p* = 0.1453). The largest, more than three-fold difference between clusters was observed for proline (346%). Following the groups identified in Figure 2, groups H_2_O_2_ > TBARS and TBARS > H_2_O_2_ were given different symbols to further analyze their arrangement within clusters showing different responses to WW. Interestingly, only seven genotypes from H_2_O_2_ > TBARS were located in cluster 2, harboring genotypes with significantly higher proline accumulation (Table 1), whereas the remaining 20 genotypes were located in cluster 1, including the genotype with most extreme total phenotypic response in the two analyzed dimensions (PHW65).

**Figure 4:**
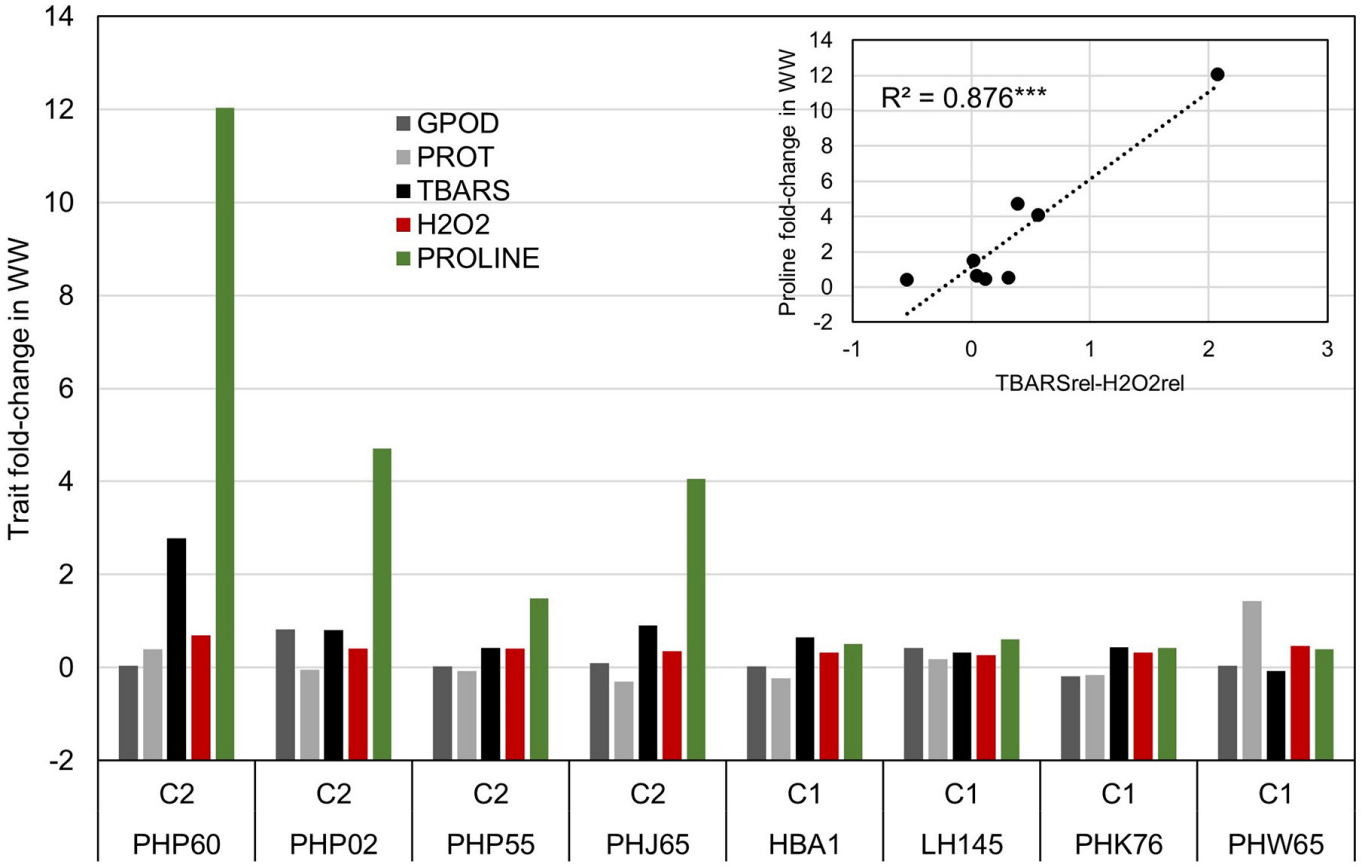
Analysis of trait fold-change values in inbred representatives surrounding the centroids of clusters in Figure 3 and the most extreme phenotypes in cluster one (PHW65) and two (PHP60). Linear model shows the relationship between the difference in trait fold-change values of TBARS and H_2_O_2_ and Proline accumulation in WW treatment in eight selected inbreds.

The selection of each cluster representatives for further analysis was carried with following heuristics. First, the three nearest-to-centroid points were selected, with centroid representing an imaginary center of cluster (average response in reduced 2d hyperplane), as cluster representatives. Second, the farthest genotype in both dimensions (PHW65 in cluster 1 and PHP60 in cluster 2) was selected. The analysis of candidate's responses (Figure 4) showed apparent differences in proline accumulation, accompanied by subtler changes in other biochemical parameters. To accompany the analysis of relationship between accumulation of H_2_O_2_ and TBARS in context of proline accumulation (Figure 2), the relative response of H_2_O_2_ was subtracted from relative response in TBARS and the simple linear regression showed highly significant relationship (*R*^2^= 0.876) between this difference and the proline accumulation (Figure 4).

### Association Analysis and Candidate Genes

**Figure 5:**
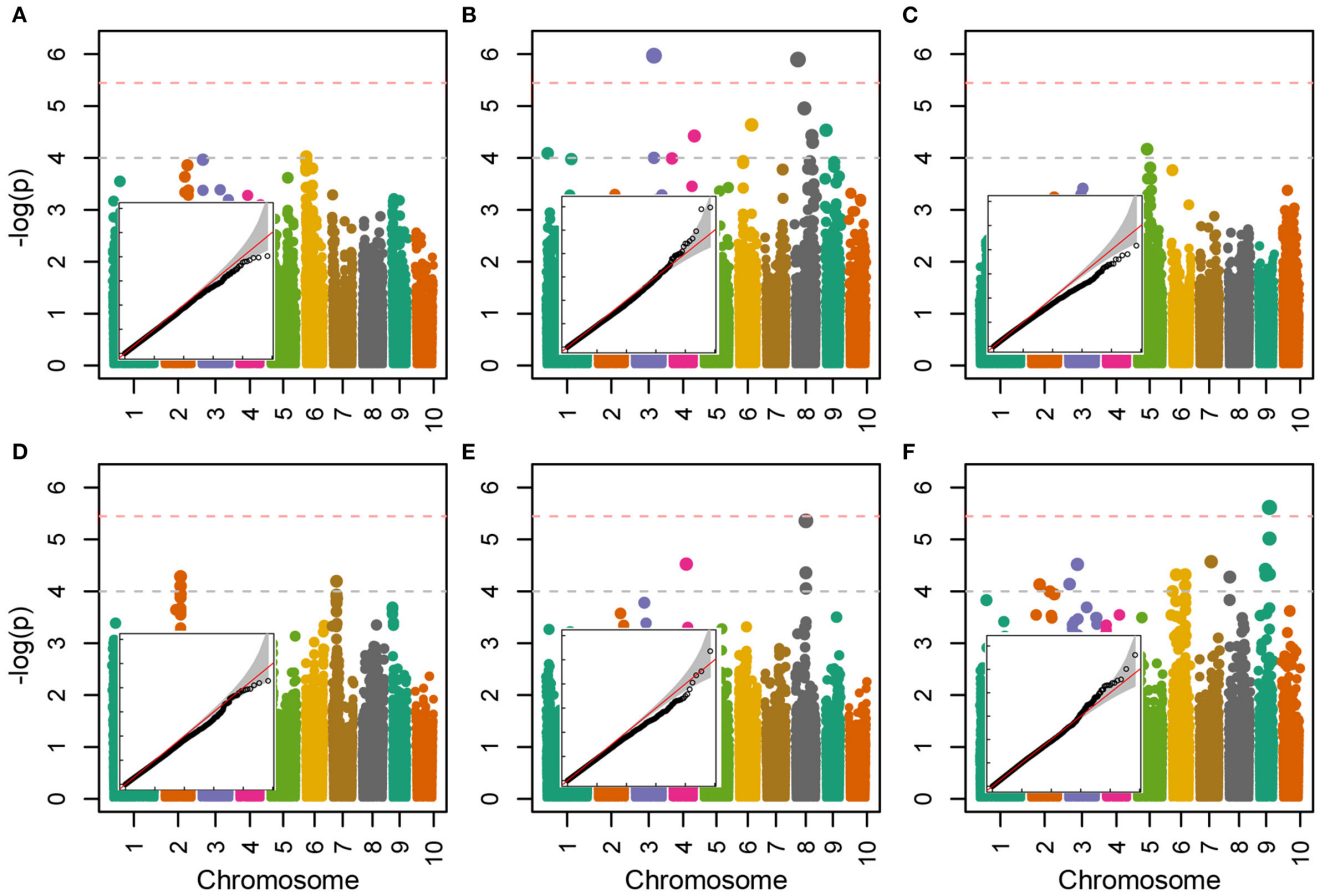
Manhattan and quantile-quantile plots of the –log(p) values of associations of the trait fold-change values between CO and WW for CWC **(A)**, proline **(B)**, H_2_O_2_ **(C)**, PROT **(D)**, GPOD **(E)** and TBARS **(F)**. Horizontal dashed lines represent arbitrary threshold of 4 (gray) and Bonferroni threshold of 5.45 (light red).

Allelic effects in association analysis in all analyzed traits followed normal distribution and no considerable deviations of effects were detected up to the value of –log(p) of 4 (Figure 5). Inflations of effects on the right tails of distributions for proline and TBARS (Figures 5B,F) indicated the presence of loci crossing the calculated Bonferroni threshold. Totally, 34 associations were declared significant (Figure 5; Table 3), most of which were detected for proline (29.4%) and TBARS (44.1%). A total of three of the 34 associations crossed the calculated Bonferroni threshold of 5.446 (Table 3), two of which were detected for proline (Figure 5B), one on chromosome three (PROLINE2@3), and another on chromosome eight (PROLINE6@8), along with a single association for TBARS, TBARS14@9 (Figure 5F). PROLINE2@3 was located on physical position 189,739,999 bp with peak –log(p) value of 5.970 and four genotypes carrying the minor variant. PROLINE6@8 was located on physical position 21,838,456 bp with peak –log(p) 5.897 and four genotypes carrying the minor allele. Single association that crossed Bonferroni threshold detected for TBARS (TBARS14@9) was located on chromosome 9, physical position 139,353,870 bp with –log(p) value of 5.616 and 12 genotypes with minor allele. Many of the variants detected as different associated loci represent same associations, however, with different numbers of genotypes carrying the minor allele. For example, associations PROT1@2 and PROT2@2 are 18.2 kbp apart with 13 and 11 minor-allele carriers. GPOD2@8 and GPOD3@8 probably also represent the same association as the distance between these loci is only 76 bp. TBARS6@6 and TBARS7@6 are 5.3 kbp apart, whereas TBARS13@9, TBARS14@9, and TBARS15@9 are 49.5 kbp and 7 bp apart, respectively. However, GPOD3@8 and GPOD4@8 might not represent the same association, as the 143.5 kbp distance exceeds the 120 kbp linkage disequilibrium block size. Within regions carrying the significant associations, 120 kbp in both directions from the peak physical locations 182 candidate genes from various metabolic pathways were found in the BioMart analysis (Supplementary Table S3) with 869 putative transcripts.

**Table 3:** Summary of detected associations for Tr_rel_ for GPOD (AU/g FW) PROT (mg/g FW), TBARS (ng/g FW), proline (nmol/mg FW), H_2_O_2_ (nmol/g FW) and CWC (%) crossing the –log(p) significance threshold of 4.

**Trait**	**Name**	**Chr**.	**Pos**.	**–log(p)**	**Candidate gene name**
CWC	CWC1@6	6	21457505	4.028	RNA-metabolizing metallo-beta-lactamase family protein
PRO	PROLINE1@1	1	9009032	4.09	beta amylase2
					Vegetative storage protein
	**PROLINE2@3**	**3**	**189739999**	**5.97**	Osmotin-like protein OSM34
	PROLINE3@3	3	189789986	4.003	NHL25
	PROLINE4@4	4	243872645	4.423	Peroxisomal membrane protein PEX16
					Probable phosphoinositide phosphatase SAC9
	PROLINE5@6	6	152847056	4.639	Coronatine-insensitive protein 1
	**PROLINE6@8**	**8**	**21838456**	**5.897**	Gigantea1
	PROLINE7@8	8	120343076	4.955	Protein NETWORKED 2D
	PROLINE8@8	8	162493033	4.433	NADPH:quinone oxidoreductase
					Protein SHI RELATED SEQUENCE 6
	PROLINE9@8	8	165274233	4.298	Delta3,5-delta2,4-dienoyl-CoA isomerase
	PROLINE10@9	9	12076699	4.535	Serine carboxypeptidase-like 29
H2O2	H2O21@5	5	74828595	4.169	Polyketide cyclase/dehydrase and lipid transport superfamily protein
PROT	PROT1@2	2	170426412	4.284	Aldo-keto reductase/ oxidoreductase
	PROT2@2	2	170444658	4.102	
	PROT3@7	7	33140990	4.194	UPF0235 protein
GPOD	GPOD1@4	4	181787137	4.524	Cyclin-dependent kinase inhibitor 1
	GPOD2@8	8	133675700	4.357	peroxidase64
	GPOD3@8	8	133675776	5.357	
	GPOD4@8	8	133819272	4.055	peroxidase2
TBARS	TBARS1@2	2	39693383	4.131	3-ketoacyl-CoA thiolase 2 peroxisomal
					Bowman-Birk type bran trypsin inhibitor
					Probable 2-oxoglutarate-dependent dioxygenase
	TBARS2@3	3	9767531	4.139	
	TBARS3@3	3	125562863	4.518	Lipid phosphate phosphatase delta
	TBARS4@6	6	17174793	4	Calreticulin-2
	TBARS5@6	6	90470962	4.319	GATA transcription factor 24
	TBARS6@6	6	154170989	4.32	Tubby-like F-box protein 10
	TBARS7@6	6	154176318	4.111	Glutathione S-transferase family protein
	TBARS8@7	7	138229143	4.571	Metallothionein-like protein 2A
	TBARS9@8	8	14999168	4.275	HSP20-like chaperones superfamily protein
					PASTICCINO 2
	TBARS10@9	9	104341296	4.424	Calcium-dependent lipid-binding (CaLB domain) plant phosphoribosyltransferase family protein
	TBARS11@9	9	110991638	4.415	Zinc finger protein CONSTANS-LIKE 5
	TBARS12@9	9	111784658	4.307	cryptochrome 3
	TBARS13@9	9	139304310	4.328	Hexosyltransferase
	**TBARS14@9**	**9**	**139353870**	**5.616**	
	TBARS15@9	9	139353877	5.016	

*In bold are the associations crossing the calculated Bonferroni threshold of 5.45. Candidate gene IDs and gene names were selected from BioMart analysis. Details of the detected associations with candidate gene IDs and their functions are available in Supplementary Table S3*.

### Gene Ontology Enrichment Analysis

**Figure 6:**
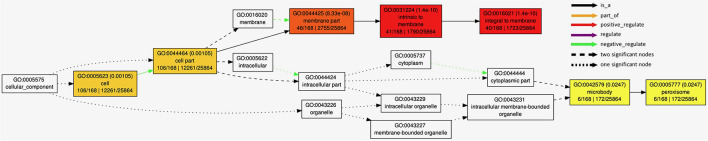
Results of AgriGo gene ontology (GO) enrichment analysis with genes detected by BioMart tool (Supplementary Table S3) for cellular components.

The GO analysis with 162 candidate genes from BioMart analysis passing the quality check with AgriGO 2.0 online mining tool showed significant enrichment of cellular components (Figure 6) and biological processes (Supplementary Figure S3). Highly significant negative regulation of the integral membrane parts was detected in cellular component analysis (Figure 6, *p* < 10^−9^), along with significant (*p* < 0.05) negative regulation of microbodies peroxisomes. Biological processes analysis (Supplementary Figure S3) showed large number of positively regulated processes (*p* < 0.05), linked to regulation of DNA-dependent transcription and polysaccharide catabolic processes.

## Discussion

### Variation of Stress-Related Traits in Water Withholding Treatment

The studies reporting quantitative genetic analysis of biochemical parameters involved in stress response are scarce, although these parameters harbor information about the well-known biological processes, such as detoxification of ROS, lipid peroxidation, or proline accumulation and might thus represent well-worth traits in breeding for osmotic stress tolerance and consequently higher yields (Tardieu, 2012). For example, in the study on sunflower hybrids by Khalil et al. (2016), higher broad-sense heritabilities were reported compared to repeatabilities detected in our study, which could be explained by the different crops, different stages and using hybrids compared to inbred lines. Several studies reported loci associated with proline accumulation in barley (Fan et al., 2015; Jang et al., 2020) and rice (Sayed et al., 2012), hydrogen peroxide build-up (Gill et al., 2019; Kumar and Nadarajah, 2020), and TBARS in rice (Jiang et al., 2009), wheat (Ma et al., 2015), and cotton (Yasir et al., 2019); however, there are no available results for these important physiological processes in maize up to this date. Non-zero genetic variances in variance component analysis (Table 1) imply feasibility of breeding directly for these traits, although the fold change compared to control might be more useful in screening of maize accessions due to the functional diversity of analyzed traits even in non-stressful conditions. Designed experiments conducted in controlled conditions lack in diverse conditions and stressors that plant must cope with in field (Farooq et al., 2012). However, any drought-related trait can confer drought tolerance if addressed to a proper climatological scenario (Tardieu, 2012), and analysis of responses to water withholding represents low-cost means for high throughput mass-screening of potentially favorable genotypes. Furthermore, it was found that the responses of maize to certain osmotic pressure were strongly correlated between controlled and field conditions (Chapuis et al., 2012).

The lowest repeatability of proline accumulation caused by the highest relative genotype by treatment interaction component is in accordance with proline manifold physiological functions and multiple pathways of biosynthesis (Verslues et al., 2014), along with differences in capacities of different genotypes to accumulate proline with increase of osmotic pressure (Khalil et al., 2016) causing the crossovers of genotype reactions. Increase in correlation strengths between different traits in WW treatment (Figure 2A) implies adaptive activation of plant physiological mechanisms to alleviate the stress effects (Bustos-Korts et al., 2018). Expectedly, reduction of plant CWC was negatively associated with TBARS, proline, and H_2_O_2_ (Figure 2B). The decrease in CWC value is expected in water deficit conditions (Avramova et al., 2016) and was shown to be negatively associated with photosynthetic efficiency and plant development in maize hybrids and antioxidant enzyme activity (Holá et al., 2017). The CWC reduction implies loss of leaf water, but without assessment of saturated leaf mass, thus providing only a loose estimate of leaf relative water content. However, its reduction is a good estimate of the effects of the drop in available soil water content especially in monitoring of drought development (Zhou et al., 2021).

### Crossovers of Genotype Reactions Indicate TBARS Signaling Function

Water withholding stress induces stomatal closure leading to impaired CO_2_ fixation and consequently excessive production of ROS such as hydrogen peroxide (H_2_O_2_) among others (Gill and Tuteja, 2010). To cope with water scarcity, plants developed different enzymatic and non-enzymatic mechanisms of ROS scavenging. Water withholding treatment caused generation of ROS in treated maize genotypes leading to increased levels of TBARS, H_2_O_2_, proline, and the activity of GPOD.

Previous studies demonstrated that H_2_O_2_ has dual role in plants by acting as a signaling molecule at low concentrations, thus triggering adaptation to stressful conditions (Gupta et al., 2016) while at higher concentrations can trigger programmed cell death (Gill and Tuteja, 2010). Its homeostasis is maintained at different cell parts and organelles such as peroxisomes by enzyme catalase (Hossain et al., 2015), in cytosol and chloroplasts by the ascorbate peroxidase (Guo et al., 2020b) and various peroxidases in mitochondria, such as guaiacol peroxidase (Tognolli et al., 2002). In this study, H_2_O_2_ levels showed induction of oxidative stress and the ROS scavenging capacity in maize genotypes exposed to water withholding stress. H_2_O_2_ is involved in numerous physiological processes in plants such as development, senescence, cell cycle, photosynthesis, and stomatal movement (Huang et al., 2019).

Peroxidases are the enzymes responsible for scavenging hydrogen peroxide and reactive intermediary forms of O_2_ under stress conditions. Guaiacol peroxidase (GPOD) is involved in oxidative stress response by catalyzing reduction of H_2_O_2_, thus decreasing its negative effects (Gill and Tuteja, 2010) using phenols (guaiacol) as a substrate. Another interesting feature of GPOD is the fact that it is involved in the process of lignification during the young plant development (Kidwai et al., 2020). It has been reported that peroxidases can play important role in ROS scavenging in maize under stressful conditions (Rohman et al., 2016), but GPOD is also a part of the antioxidant system involved in stress-acclimation resulting in transcriptional cell modifications (Gechev et al., 2006). Increase of peroxidase activity reduces ROS accumulation and also has the ability to consequently regulate the level of lipid peroxidation (Huang et al., 2019) to some extent.

Lipid peroxidation in parallel occurs in healthy organisms and is carried out both enzymatically (lipoxygenase activity) and non-enzymatically (ROS-mediated) (Farmer and Mueller, 2013), and thus, the change in TBARS accumulation in sub-optimal conditions is of true interest for understanding of trait implications. The activation of lipid peroxidation mechanism is characterized by three stages that include initiation, progression, and termination when ROS levels reach threshold on the cellular level, thus initiating production of lipid-derived radicals. Oxidation of lipids in the cell membranes leads to its instability by decrease of membrane fluidity and increase of membrane leakage which consequently inactivates different transport mechanisms such as receptors, ion channels, and enzymes (Gill and Tuteja, 2010). Rohman et al. (2016) reported higher MDA levels in maize seedlings more susceptible to drought stress while Zhang et al. (2014) showed that after exposure of maize seedlings to sudden drought stress, MDA levels were more increased in comparison with gradual drought stress. Although the increase in MDA values is usually interpreted in terms of stress damage, it was found that increase in lipid peroxidation can also shift the transcriptional profile of a cell (Weber et al., 2004). Furthermore, recently, it was speculated that the MDA might serve as a stress-signaling molecules in plants activating dehydration/heat shock-related genes and genes involved in antioxidant machinery (Morales and Munné-Bosch, 2019).

Accumulation of osmolytes, such as proline, under drought stress was reported in various plant species. Proline is a member of the glutamate family and plays versatile role in maintaining water status, membrane stability, inhibiting protein oxidation and ROS scavenging under osmotic stress, thus contributing to drought-stress tolerance (Hayat et al., 2012). Genotypes exhibiting higher proline accumulation in water deficit are considered to be more drought tolerant (Gill and Tuteja, 2010; Ozturk et al., 2021). Yang et al. (2009) demonstrated that in maize seedlings, H_2_O_2_ could be involved in mechanisms of signal transduction that can lead to proline accumulation as well as regulation of its biosynthesis and degradation. Namely, proline accumulation induced by H_2_O_2_ is formed *via* combination of consecutive activation of the two biosynthetic pathways and inhibition of the proline degradation by H_2_O_2_. This was also confirmed in our study by the moderate positive significant correlation between proline and H_2_O_2_ in WW (Figure 2A) as well as their respective changes in WW compared to CO (Figure 2B).Our analysis of fold-changes between CO and WW indeed confirmed the relationship between the H_2_O_2_ build-up and proline accumulation as the top 10 scorers for H_2_O_2_ change in WW showed more than 2-fold increase in proline accumulation (Figure 2C). However, the top 10 scorers in TBARS accumulation showed even larger, more than 4-fold increase in proline accumulation. The division of the inbreds to two groups with fold change of H_2_O_2_ > TBARS (*n* = 27), and TBARS > H_2_O_2_ (*n* = 82) further confirmed the observed pattern. This finding is in accordance with the assumed signaling functions of MDA in the activation of heat shock and dehydration-related genes (Morales and Munné-Bosch, 2019), especially since there is a known relationship between heat shock proteins and proline accumulation in osmotic stress conditions (Augustine, 2016).

### K-Means Clustering, Association Analysis, and Gene Ontology Enrichment Corroborate the Interplay Between H_2_O_2_ and TBARS in Osmotic Regulation

K-means clustering (Figure 3) showed that the assessed genotypes can be readily divided into two clusters of reactions to WW: one with larger change in trait values in WW compared to CO and another with lower change (Table 2) indicating the feasibility of division of cultivars to drought sensitive and drought tolerant (Gill and Tuteja, 2010) at this growth stage. Due to the lack of link between cluster designations and available pedigree and admixture data, K-means cluster affiliations were used as covariate to control FDR, thus compensating for relatively modest sample size (Aschard et al., 2017). Interestingly, cluster 2 was mostly populated by inbreds from TBARS > H_2_O_2_ group, bearing only seven accessions of the H_2_O_2_ > TBARS group, scattered near zero at both dimensions. This corroborated our speculation of the involvement of TBARS in proline accumulation, as inbreds belonging to cluster 2 showed 3.4 times increase in proline content in WW treatment, and 54.2% larger increase in TBARS compared to inbreds in cluster 1. It is also worth mentioning that the accession (PHW65) with the most extreme phenotype considering all analyzed traits in cluster 1 belonged to H_2_O_2_ > TBARS group, whereas in cluster 2, inbred PHP60, showing the most extreme phenotype, belonged to the group TBARS > H_2_O_2_. Noteworthy, the three nearest accessions to the centroid of the cluster 2, as well as the most extreme phenotype of cluster 2, belong to the Iodent breeding pool, namely, PHP60, PHP55, PHP02, and PHJ65, while closest to the centroid of the cluster 1, the inbreds HBA1 and LH145, belonging to Lancaster and B14 breeding pools, are found. Iodent group represents a relatively novel germplasm pool, which was not commercially utilized prior to 1980s (Mikel and Dudley, 2006) and might thus be associated with the genetic variability underlying the detected signals, especially as the more recent breeding efforts were inclined toward drought tolerance (Troyer, 2009). The Iodent breeding pool might thus indeed carry the favorable alleles for osmotic stress tolerance. This was confirmed in a recent study on 209 diverse inbred lines at seedling stage, where Iodent pool founder line, PH207, was selected among the four most drought tolerant accessions (Guo et al., 2020a). It is worth to emphasize that all except one inbred (PHW65) in the Figure 4 belong to TBARS > H_2_O_2_ group, so the involvement of lipid peroxidation—hydrogen peroxide crosstalk cannot be unambiguously pinpointed as a sole contributor to the proline accumulation. However, inspection of the regression results in this group of inbreds, with very high coefficient of determination between difference between H_2_O_2_ and TBARS fold change in water withholding and proline accumulation, along with other aforementioned patterns, indicates possible important role of this pathway in osmoprotection of young plants.

Details on the associations detected in association analysis (Figure 5; Table 3) are available in Supplementary Table S2 available online, along with most probable gene candidates identified from full gene list from BioMart (Kinsella et al., 2011) scan (Supplementary Table S3). The genes identified in BioMart were used in AgriGo online mining tool (Tian et al., 2017) for the gene ontology enrichment analysis. The highly significant negative regulation of intrinsic membrane parts revealed from genes detected in association analysis in GO enrichment analysis (Figure 6) was probably caused by the damage to the cell membrane causing membrane lipid-bilayer distortion. The distortion of the lipid-bilayer affects the functions of the surrounded proteins, causing the alteration of their functionality (Lee, 2004). The detected enrichment was supported by the involvement of 40 detected genes in gene network regulating these components. Other negatively regulated cell components were peroxisomes. Interestingly, H_2_O_2_ is a well-known stress signaling molecule, with peroxisomes being their main source (Hossain et al., 2015; Su et al., 2019). It is possible that the negative regulation of the resource of this important signaling molecule represents the part of the signaling cascade causing the differences in proline accumulation between groups observed in Figures 2B,C. This is further corroborated by the rates of accumulation of H_2_O_2_ in organelles. For example, the peroxide accumulation in mitochondria does not show large variation throughout the day, whereas the rates of its formation can be 30 to 100 times higher in chloroplasts and peroxisomes (Hossain et al., 2015). Furthermore, H_2_O_2_ synthesis in peroxisomes is associated with photorespiration, or, more specifically, oxidation of glycolate during the photosynthetic carbon oxidation cycle (Niu and Liao, 2016), impacting the gene expression and metabolic enzyme activity. The crosstalk in proline synthesis between lipid peroxidation and H_2_O_2_ is further corroborated by the genes linked to associations TBARS10@9 and TBARS13@9-TBARS15@9, coding for calcium-dependent lipid-binding (CaLB domain) plant phosphoribosyltransferase family protein and hexosyltransferase, both acting as glycosyl transferases in our study (Supplementary Table S2). On the other hand, the study by Ben Rejeb et al. (2015) showed that accumulation of the H_2_O_2_ generated by the NADPH oxidases in *Arabidospis thaliana* plants increases the proline biosynthesis by positive regulation of proline biosynthesis genes. Interestingly, the association detected in our study (PROLINE8@8) was located in the region harboring the NADPH quinone oxidoreductase linked to NADPH oxidation, also involved in the detoxication process of lipid peroxides (Mano et al., 2002), possibly representing another link between the TBARS and proline accumulation. Also, the dehydration response genes were detected, such as OSM34 coding for osmotin, related to association PROLINE2@3. Osmotines are plant sentinel proteins expressed during the osmotic stress, providing plant cell the means to retain osmolarity by metabolic changes and solutes compartmentalization (Ozturk et al., 2021), such as proline. There is abundant evidence that the expression of osmotin genes triggers proline accumulation in osmotic stress in many species (for review, see Anil Kumar et al., 2015). Other genes that possibly corroborate the relationship assumed by Morales and Munné-Bosch (2019) that the MDA might act as signaling molecules in stress are heat shock-related genes such as HSP20 linked to association TBARS9@8 (Figure 5; Table 3). It was found that the heat shock proteins, acting as chaperones, are readily expressed in osmotic stress conditions, helping in binding, folding, displacing, and degrading other proteins (Ozturk et al., 2021). Furthermore, there is a known crosstalk between heat shock proteins and proline in heat stress, mediated by nitric oxide (Alamri et al., 2019), possibly active in osmotic stress as well.

Most interestingly, the correlation analysis (Table 2) showed the lower correlation between TBARS and H_2_O_2_ in TBARS > H_2_O_2_ group compared to correlation observed in H_2_O_2_ > TBARS, possibly implying the enzymatic rather than ROS-mediated origin of TBARS (Farmer and Mueller, 2013). This was accompanied by the increase in correlation strength between TBARS and proline in TBARS > H_2_O_2_ group implying the activation of additional proline synthesis mechanisms. The H_2_O_2_ and lipid peroxidation homeostasis might thus play a critical role in proline synthesis. In a study by Terzi et al. (2014), it was shown that H_2_O_2_ treatment in maize affects levels of proline and MDA in leaves in osmotic stress conditions. The process might be mediated through activation of polysaccharide catabolic processes, as proline synthesis is an energy-consuming task. The differentially regulated polysaccharide catabolism (Supplementary Figure S3) covered the gene related to detected association PROLINE1@1 (Table 3) coding for beta-amylase2, and the beta amylases are known for their role in providing energy for proline synthesis in drought stress (Zanella et al., 2016). Finally, it was found that 24 of 168 genes analyzed in AgriGo tool were involved in significant enrichment of regulation of DNA-dependent transcription, causing the transcriptional reprogram of cells.

In conclusion, the reactions of inbred lines assessed in this study allowed detection of a potentially significant regulatory signaling mechanism for response to water withholding at young plant growth stage. Namely, the results of this mass-screening indicate that in response to water withholding, the group of inbreds in which accumulation of products of lipid peroxidation (TBARS) surpassed the accumulation of H_2_O_2_, a well-known signaling molecule, in average increased their proline content by nearly a double. Furthermore, the analysis of inbred responses to water withholding per se corroborated by the K-means cluster analysis showed lower variability in all assessed biochemical traits in H_2_O_2_ > TBARS group accompanied by nearly no change in proline content. For example, the representatives of the two K-means clusters showed radical differences in proline accumulation, where inbred PHP60 (cluster 2, TBARS > H_2_O_2_) was able to increase proline content nearly 12-folds in WW, whereas inbred PHW65 (cluster 1, H_2_O_2_ > TBARS) produced barely detectable response to osmotic changes detectable by the used methods. The association mapping combined with gene ontology enrichment analysis showed significant phenotypic effects of the linkage regions harboring genes involved in osmotic-stress signaling and osmolyte accumulation, as well as negative regulation of peroxisomes, corroborating the phenotypic analysis. However, the contrasting responses to water withholding of the two groups of inbreds in this study do not necessarily reflect to the final (agronomic) performance of the genotypes, so three important aspects have to be elucidated in further research to establish the implications of these findings: (i) the dynamic analysis in genotypes with contrasting responses (e.g., top 10 and bottom 10 TBARS scorers) needs to capture the switching of this mechanism, and the difference in dynamics of proline accumulation between the groups; (ii) the transcriptome of known regulatory genes, and the implications of this cascade through the developmental cycle haves to be studied, and (iii) the field trials in relevant environments need to provide the connection between the contrasting performance at early growth and agronomic performance in terms of yield quantity and quality.

## Data Availability Statement

The datasets presented in this study can be found in online repositories. The names of the repository/repositories and accession number(s) can be found in the article/Supplementary Material. Genomic data for the analyzed accessions is available as supplementary data at: https://doi.org/10.3390/plants9020275.

## Author Contributions

VG, SM, LB, and DŠ conceived the study and prepared the first manuscript draft. VG, ZZ, AB, and MMaz conducted the experiments. SM, MMar, and LB performed laboratory analyses. VG analyzed results. ZZ and DŠ acquired funding. All authors contributed to the article and approved the submitted version.

## Funding

This research was funded by the EU project Biodiversity and Molecular Plant Breeding, Grant Number KK.01.1.1.01.0005, of the Centre of Excellence for Biodiversity and Molecular Plant Breeding (CroP-BioDiv), Zagreb, Croatia.

## Conflict of Interest

The authors declare that the research was conducted in the absence of any commercial or financial relationships that could be construed as a potential conflict of interest.

## Publisher's Note

All claims expressed in this article are solely those of the authors and do not necessarily represent those of their affiliated organizations, or those of the publisher, the editors and the reviewers. Any product that may be evaluated in this article, or claim that may be made by its manufacturer, is not guaranteed or endorsed by the publisher.
