# Supplementary material for: Contrasting Water Withholding Responses of Young Maize Plants Reveal Link Between Lipid Peroxidation and Osmotic Regulation Corroborated by Genetic Analysis
Source: Front Plant Sci. 2022 Jul 6;13:804630. doi: 10.3389/fpls.2022.804630 (PMC9296821; doi:10.3389/fpls.2022.804630)
Supplement: Supplementary file 3 [file Data_Sheet_2.docx]

Supplementary Material

## Supplementary Figures


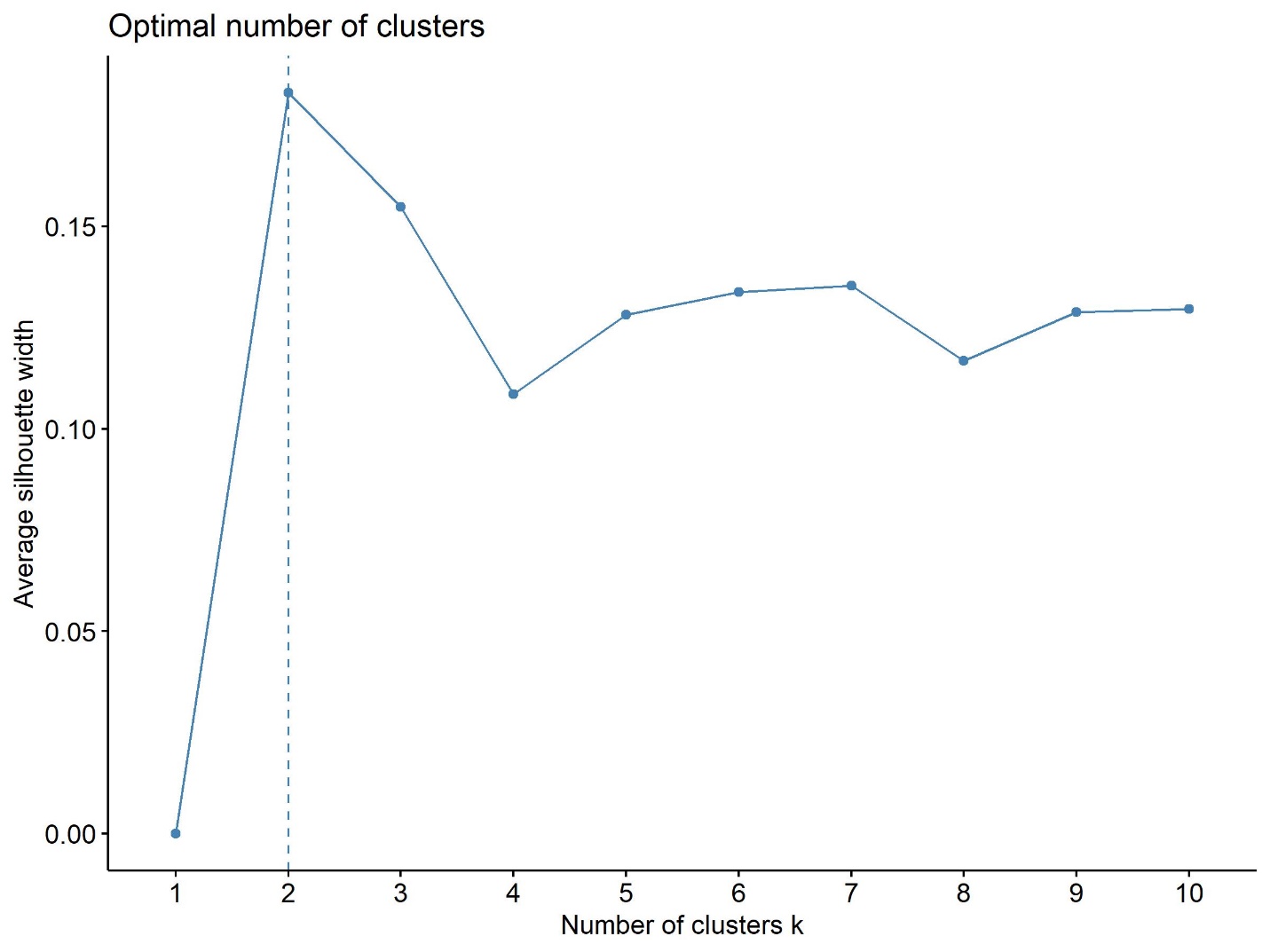


**Supplementary figure 1**. Silhouette score for optimal number of clusters in K-means analysis.


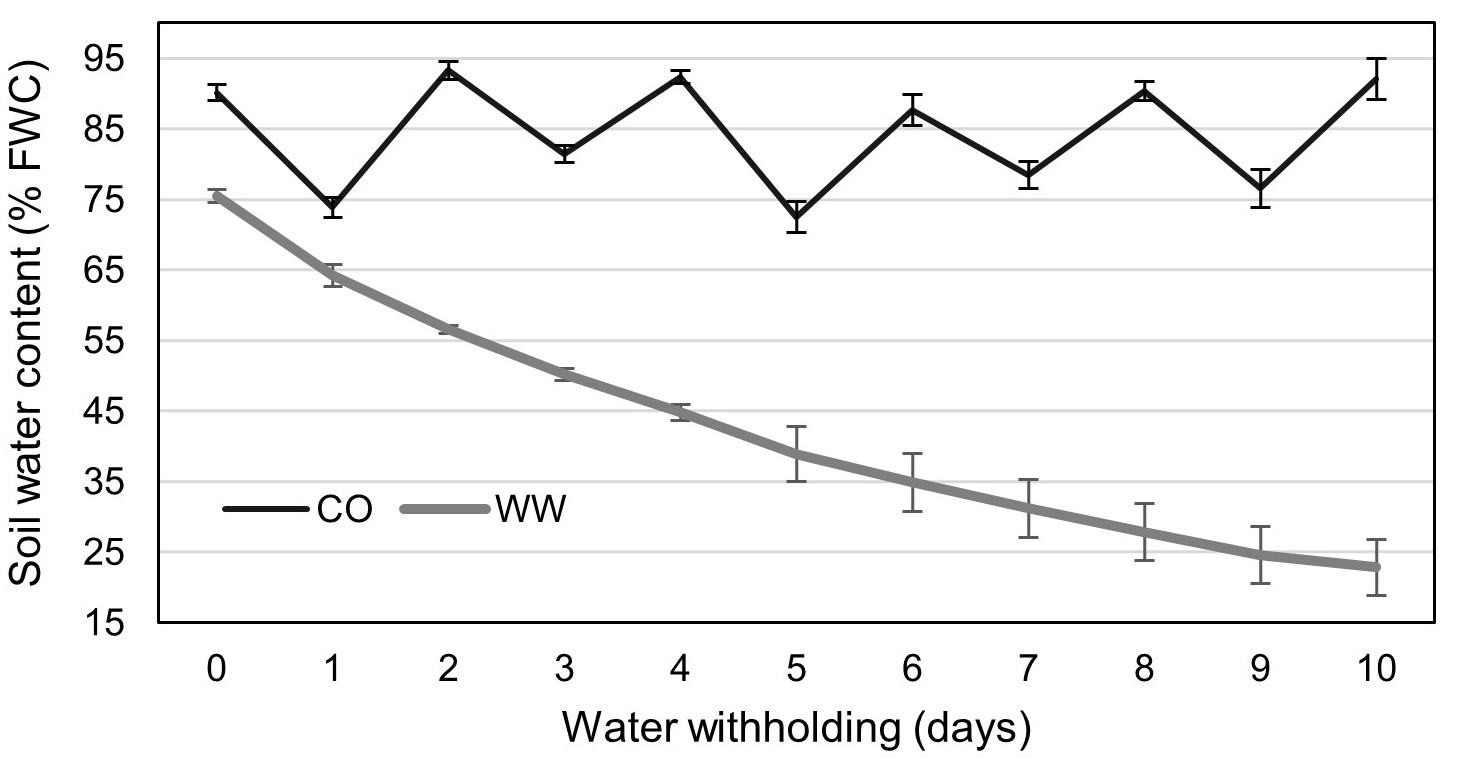


**Supplementary figure 2**. Soil water content ± standard deviation (n=4) in Control (Co) and water withholding treatment (WW) expressed as % of field water capacity (FWC) since the day of last watering (half a dose in WW, full dose in Co) until the plant tissue collection (day 10 of WW).


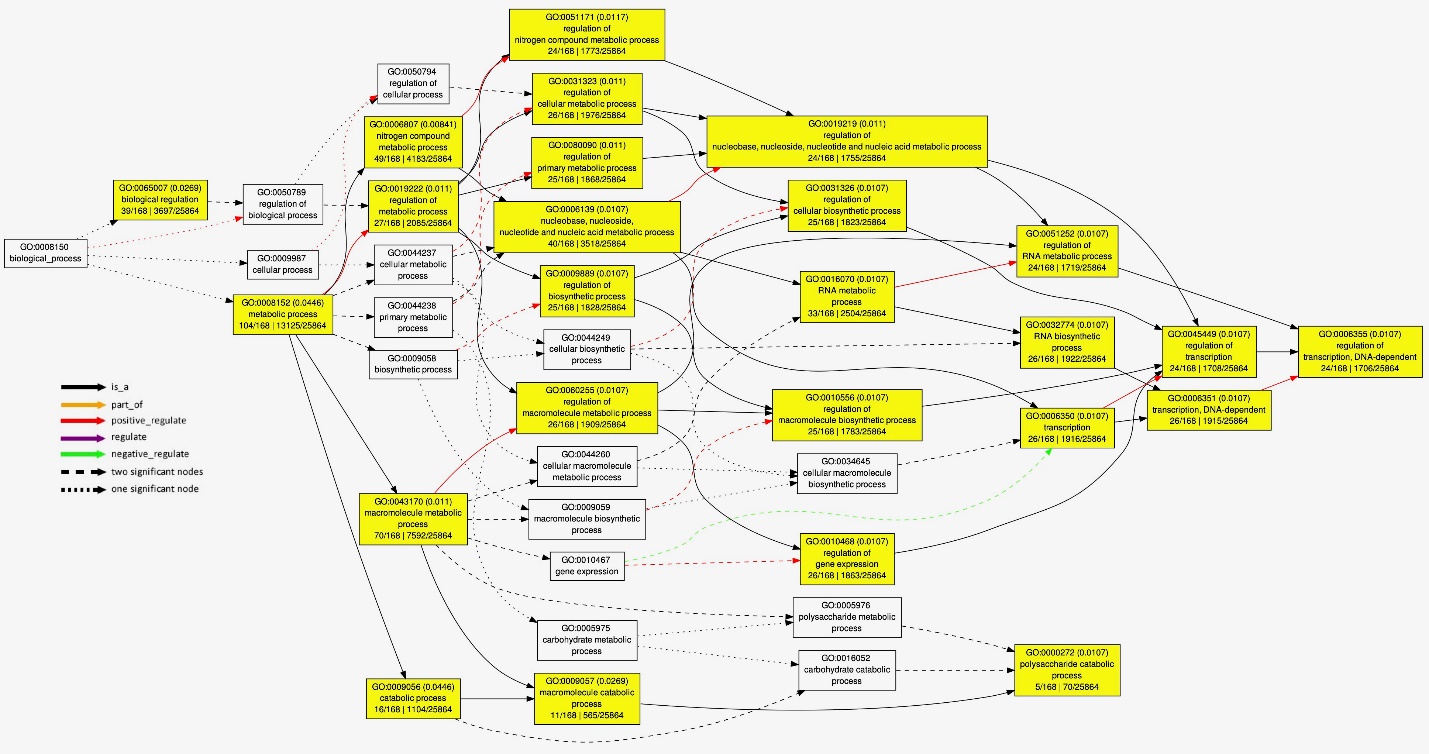


**Supplementary figure 3**. Results of AgriGo gene ontology enrichment analysis with genes detected by BioMart tool (Supplementary table 2) for biological processes
